# Ipsilateral Double May-Thurner Syndrome With a Congenital Double Left Common Iliac Vein: A Case Report of an Unprecedented Anatomical Variant and Hemodynamic Interaction

**DOI:** 10.7759/cureus.74633

**Published:** 2024-11-27

**Authors:** Roel Meeus, Pavell Dhondt, Stan Beunens, M. Akram Khan

**Affiliations:** 1 Cardiology, KU Leuven, Leuven, BEL; 2 Cardiology, Cardiac Center of Texas, McKinney, USA

**Keywords:** congenital variant, may-thurner syndrome, pelvic venous insufficiency, rare case, venous compression syndromes

## Abstract

This case report describes a unique presentation of May-Thurner syndrome (MTS) in a 28-year-old woman, characterized by the congenital bifurcation of the left common iliac vein (LCIV) into the outer (OLCIV) and inner (ILCIV) segments. Both veins experienced significant compression - OLCIV proximally and ILCIV medially - due to the overlying right common iliac artery (RCIA) and lumbar spine. The patient presented with bilateral spider veins, lower extremity swelling, pelvic discomfort, and bilateral leg cramping. Detailed imaging with venous duplex ultrasound, venography, and intravascular ultrasound (IVUS) revealed not only chronic venous insufficiency in the greater saphenous veins but also this rare vascular anomaly. Interventional treatment involved the strategic deployment of two stents to address the dual venous compression. A 16 mm x 90 mm stent was placed in the OLCIV at the site of proximal compression, followed by the insertion of a 16 mm x 60 mm stent in the ILCIV to relieve the medial compression. The successful resolution of venous obstruction was confirmed by post-procedural venography and IVUS. This case underscores the critical role of comprehensive anatomical evaluation, including advanced imaging techniques, in the diagnosis and management of complex venous disorders. The effective use of stenting in this rare “double May-Thurner syndrome” demonstrates the potential for targeted endovascular interventions in similarly challenging cases.

## Introduction

Vascular compression syndromes are rare medical conditions affecting less than 1% of the population, primarily manifesting in young individuals. These conditions occur when blood vessels are constricted by adjacent structures, leading to symptoms such as arterial ischemia, embolism, venous stasis, thrombosis, and hematuria. The severity and nature of symptoms depend on the specific vessels involved. Various arterial and venous compression syndromes include thoracic outlet syndrome, Paget-Schroetter syndrome, quadrilateral space syndrome, median arcuate ligament syndrome, nutcracker syndrome, May-Thurner syndrome (MTS), and popliteal entrapment syndrome [1].

One of the notable vascular compression syndromes is MTS, also known as iliac vein compression syndrome, which involves the compression of the left common iliac vein (LCIV) by the overlying right common iliac artery (RCIA) and the fifth lumbar vertebra. This compression can lead to iliac vein or iliofemoral venous thrombosis, resulting in a range of symptoms primarily affecting the left leg. First observed by Virchow in 1851, who noted a left-sided predominance of iliofemoral deep vein thrombosis (DVT), May et al. later provided a detailed anatomical description of this phenomenon [2]. Although the exact prevalence of MTS is unclear due to many asymptomatic cases, it is estimated to occur in 2-5% of patients with venous disease. A study by Kibbe et al. found that 24% of an asymptomatic population showed signs of MTS through CT imaging. The syndrome predominantly affects women, with a female-to-male incidence ratio of 2:1, and is most common between the ages of 20 and 40 [3,4].

In this article, we present a unique and unprecedented case of an "ipsilateral double May-Thurner" syndrome resulting from the congenital splitting of the LCIV into an outer and inner LCIV, with both of them being compressed by the RCIA.

This article was previously presented as a poster presentation at the VENOUS2024 conference on March 5, 2024.

## Case presentation

A 28-year-old woman of Eastern European descent, with no significant medical history besides active oral contraceptive use, presented with bilateral spider veins on the lower extremities, bilateral lower limb edema, constant dull pelvic discomfort exacerbated during intercourse, and bilateral leg cramping and heaviness, particularly worsening in the evening. Despite initial conservative therapy, her symptoms persisted, leading to a referral to a vascular specialist.

Upon thorough physical examination, bilateral swelling in the extremities, varicose veins in the left and right lower extremities, bilateral telangiectasias, and 4-5 mm edema on both sides were noted. Bilateral pitting edema was present, while signs of hemosiderin staining, erythema, ecchymosis, and open ulcers were absent. A venous duplex ultrasound confirmed a diagnosis of clinical class III varicose veins and chronic venous insufficiency (CVI), predominantly affecting the greater saphenous veins bilaterally, with chronic venous reflux disease causing pain and swelling in the lower extremities.

**Figure 1:**
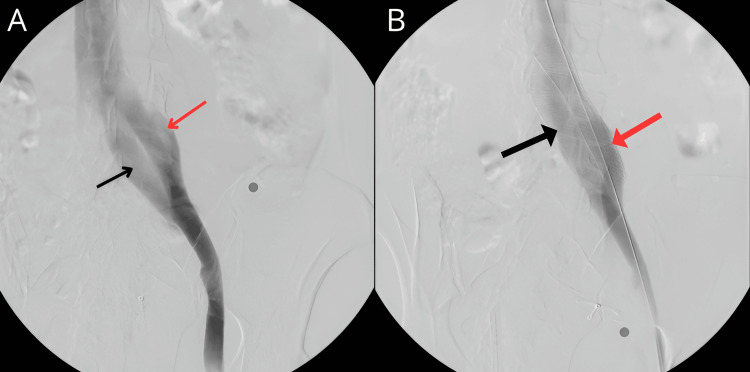
Venogram of both the outer (thin red arrow) and inner (thin black arrow) left common iliac veins before double stent placement (A) and venogram of the outer (thick black arrow) and inner (thick red arrow) left common iliac veins after double stent placement (B).

Due to concomitant pelvic symptoms, further assessment was warranted to exclude MTS. A venogram and intravascular ultrasound (IVUS) were performed. The procedure involved gaining venous access through the right femoral vein, followed by the careful advancement of a 0.35" microwire in a retrograde manner to reach the LCIV. The venogram revealed a rare anatomical variant: a unique bifurcation of the LCIV into an outer LCIV and an inner LCIV, stemming from congenital splitting at lumbar level L5 (Figure 1).

**Figure 2:**
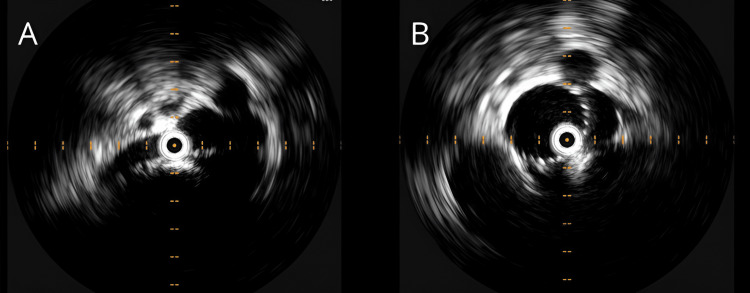
IVUS of the outer left common iliac vein before stent placement (A) and IVUS of the outer left common iliac vein after stent placement (B).

**Figure 3:**
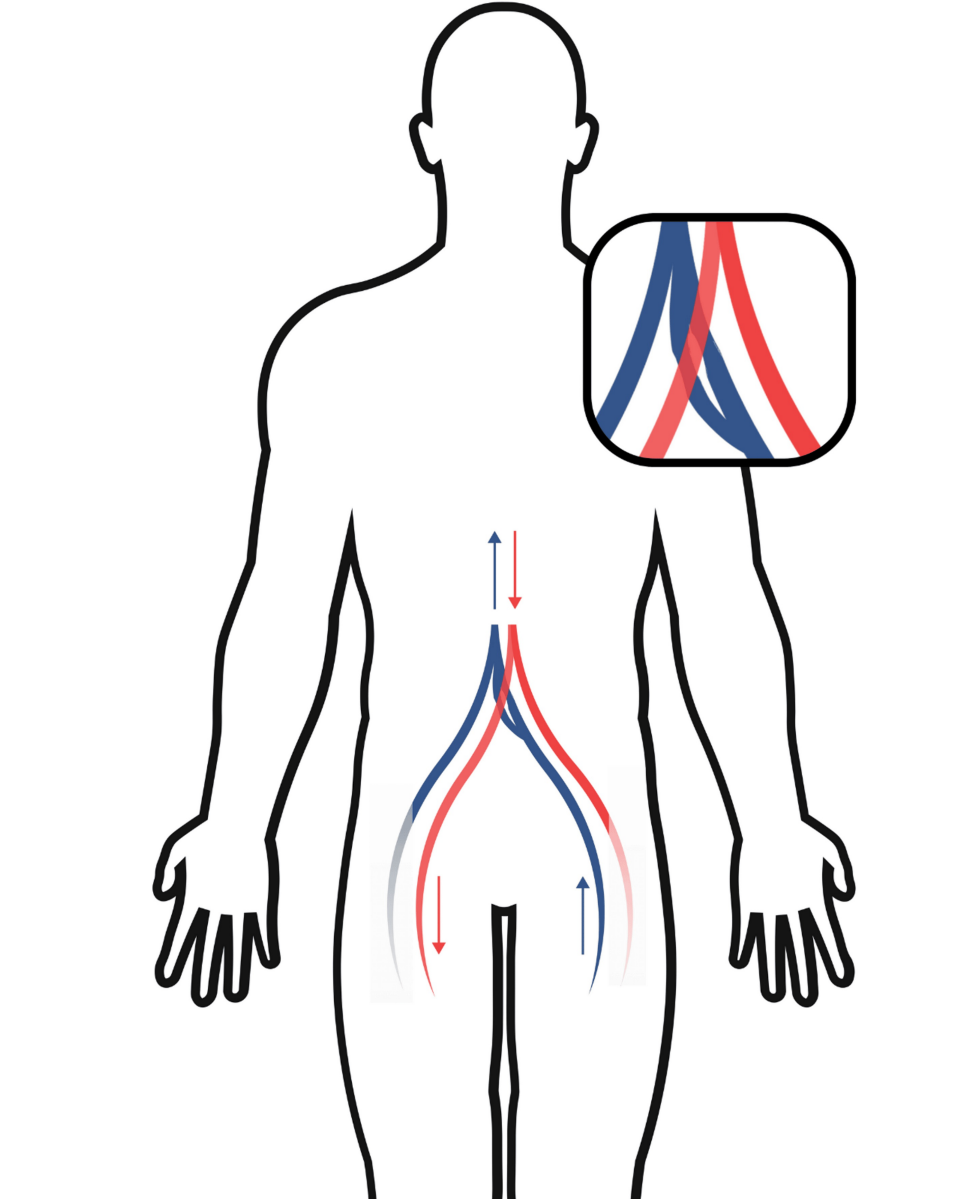
Visual representation of the anatomical interaction. The right common iliac artery overlies both the outer left common vein and the inner left common iliac vein. The outer left common iliac vein is compressed proximally, and the outer right common iliac vein is compressed centrally.

IVUS demonstrated compression of both variant veins by the RCIA, with the outer LCIV compressed in the upper third segment and the inner LCIV compressed in the middle third segment, attributed to the anatomical configuration of the RCIA (Figure 2). Thus, the patient was diagnosed with ipsilateral double MTS. A visual representation of the anatomical interaction is also shown in Figure 3.

Subsequently, a 16 mm × 90 mm stent (Venous WALLSTENT, Boston Scientific, Natick, MA) was deployed across the lesion in the upper third segment of the outer LCIV, followed by the placement of a second 16 mm × 60 mm stent in the middle third segment of the inner LCIV. The different locations of stent placement were determined by the varying sites of anatomical compression due to the RCIA. Surgical repair was not considered an option as this patient was treated in an outpatient center. Successful repair of venous compression was achieved, with no complications documented at follow-up.

## Discussion

A comprehensive understanding of embryological vascular development is essential for elucidating the origins of vascular anomalies. During the fifth week of embryogenesis, the venous system initiates formation, giving rise to the cardinal veins, which play a critical role in draining the embryonic body. These cardinal veins eventually differentiate into the anterior and posterior cardinal veins, which drain the cephalad portion and the remaining parts of the embryo, respectively. Between the sixth and tenth weeks of gestation, the posterior cardinal veins evolve into the iliac bifurcation and iliac veins. Therefore, malformations in this developmental phase can lead to variations in the hypogastric venous drainage system, as reflected in the clinical presentation of such anomalies [5].

Venous variations are not uncommon, and the iliac venous system is no exception. Documented anatomical variations in the CIV include its absence, duplication, and fenestration [2-6]. Duplication of the CIV, however, is exceedingly rare, occurring in only 0.2% of cases [5]. These variations underscore the complexity and potential challenges in diagnosing and managing related venous disorders.

CVI represents a significant clinical challenge, arising when lower extremity veins fail to efficiently return blood to the heart. This venous dysfunction leads to sustained high pressure within the veins, resulting in symptoms such as leg cramping, heaviness, fatigue, and skin changes. While CVI is often linked to common etiologies such as congenital valve defects, prior deep vein thrombosis (DVT), or biochemical changes affecting venous valves, rare conditions such as MTS must be considered, particularly when patients present with concurrent pelvic congestion symptoms [7].

The classical MTS as originally described has the left-sided MTS that concurs with the anatomical route of the right common iliac artery. There have been multiple anatomical variants described such as the compression of the right CIV but also variants in the compressing artery, such as the left internal iliac artery [8]. Other case reports detailing anatomical variants have talked about the occurrence of a “double May-Thurner syndrome” encompassing variants such as a double stenosis of LCIV caused by both RCIA and LCIA or a right CIA aneurysm causing compression of bilateral CIVs [9,10]. Further reports also detailed the pathophysiology for the increased risk for other venous compression syndromes in patients with underlying MTS [11].

What distinguishes this case is the unprecedented presentation of an "ipsilateral double May-Thurner syndrome," resulting from a congenital splitting of the LCIV with subsequent dual compression by the RCIA. To our knowledge, this is the first reported case of such a dual pathology. The patient presented with bilateral spider veins, cramping, and lower leg edema, yet without evidence of pulmonary embolism or DVT. This highlights the importance of considering rare anatomical variations in the differential diagnosis of CVI, particularly in patients with atypical presentations.

The management of this complex venous pathology focused on addressing the stenotic lesions through endovascular intervention. Given the high recurrence rates associated with angioplasty alone, the decision was made to proceed with stenting. The use of self-expanding stents, followed by balloon dilation, proved crucial in achieving full expansion and reducing the risk of restenosis. Extending the stent into the inferior vena cava (IVC) did not result in adverse effects, corroborating existing literature on the safety and efficacy of this approach in treating MTS [12].

This case underscores the critical importance of detailed anatomical knowledge and the use of advanced diagnostic tools such as venography and IVUS in the management of complex venous diseases. The identification and treatment of such rare venous anomalies are vital, not only for improving patient outcomes but also for advancing our understanding of vascular pathophysiology. Further research and documentation of similar cases are necessary to refine our diagnostic and therapeutic strategies, ensuring that patients with rare vascular conditions receive the most effective care.

## Conclusions

This case report documents the first known instance of an ipsilateral double MTS, a rare and complex venous anomaly resulting from congenital splitting of the LCIV and subsequent dual compression by the RCIA. It underscores the critical importance of comprehensive anatomical assessments, including venography and IVUS, in both the diagnosis and management of complex venous pathologies. The successful utilization of stent placement, in this case, highlights its effectiveness as an intervention to relieve venous compression in such rare vascular variations. The insights gained from this case contribute to the growing body of knowledge on venous anomalies and their treatment, emphasizing the need for continued research in this area.
